# Amebicidal effects of fenugreek (*Trigonella foenum‐graecum*) against *Acanthamoeba* cysts

**DOI:** 10.1002/fsn3.849

**Published:** 2019-01-28

**Authors:** Yasemin Kaya, Ayşe Baldemir, Ülkü Karaman, Nilay Ildız, Yeliz Kasko Arıcı, Gamze Kaçmaz, Zeynep Kolören, Yusuf Konca

**Affiliations:** ^1^ Faculty of Medicine Department of Internal Medicine Ordu University Ordu Turkey; ^2^ Faculty of Pharmacy Department of Pharmaceutical Botany Erciyes University Kayseri Turkey; ^3^ Faculty of Medicine Medical Parasitology Department Ordu University Ordu Turkey; ^4^ Faculty of Pharmacy Department of Pharmaceutical Microbiology Erciyes University Kayseri Turkey; ^5^ Faculty of Medicine Biostatistics Department Ordu University Ordu Turkey; ^6^ Faculty of Medicine Medical Microbiology Department Giresun University Giresun Turkey; ^7^ Department of Biology Ordu University Faculty of Arts and Sciences Ordu Turkey; ^8^ Faculty of Agriculture Department of Feed and Animal Feeding Erciyes University Kayseri Turkey

**Keywords:** *Acanthamoeba castellanii*, amebicidal effect, fenugreek, *Trigonella foenum‐graecum*

## Abstract

*Trigonella foenum‐graecum* L. *(TF)* is known to the public as a chest emollient, mucous expectorant, laxative and is used to prevent maturation of boils and diabetes since ancient times. In this study, we aimed to determine the amebicidal effects against *Acanthamoeba* cysts. Plant extracts were prepared at concentrations of 1, 2, 4, 8, 16, and 32 mg/ml and were placed in a hemocytometer with cell counts 22 × 10^6^ cell/ml. The fatty acid profiles of *TF* seeds were determined. Standard *Acanthamoeba* cysts were added and incubated at 25°C. The viability of the parasite was checked and recorded at hours 3, 24, 48, 72, 96, and 102. The values of lethal concentration doses (LD50 and LD90) were calculated using probit analysis. This study revealed that *T. foenum‐graecum* prevented proliferation of the parasite at certain times. However, further for in vivo and controlled experimental studies are needed in order to find out how to use this plant as medication.

## 1 INTRODUCTION

Protozoa are small microscopic single‐cell organisms that usually live in liquid environment. They have a nature, which can perform all life events independently. Therefore, functions of the organs are fulfilled by the organelles in a single cell in metazoa that are the most developed cells. Most protozoa live freely in nature, but there are also metazoa species that spend a part or all of their lives in another organism. These are active cells with a distinct cytoplasm and nucleus. The cytoplasm involves glycogen granules. It can harbor liquid as well as solid nutrients. There are both organic and inorganic substances in the cytoplasm (Demirsoy, 1998; Mandell, Douglas, & Bennett, 2000; Murray, Rosenthal, Kobayashi, & Pfaller, 2002).

Although there are variations, bacteria are usually smaller than protozoa. Their body structure is homogeneous, consisting of cell membrane and cytoplasm. They have no distinct nucleus, and the chromatin is dispersed within the cytoplasm. Bacteria do not contain glycogen, they reproduce by dividing into two, replicate more compared to protozoa, and have autotrophic nutrition (Demirsoy, 1998; Mandell et al., 2000; Murray et al., 2002).

*Acanthamoeba* is among protozoa that have a highly developed structure compared to bacteria. *Acanthamoeba* is an ameba species that are freely living in natural water resources, sea water, and soil (Saygı & Polat, 2003; Sharma, Garg, & Rao, 2000). This organism has also been isolated from various environments such as swimming pool, tap water, and bottled mineral water, and even in contact lens maintenance water (Saygı & Polat, 2003; Sharma et al., 2000). *Acanthamoeba* is an opportunistic pathogen and has trophozoite and cyst forms. The trophozoite form lives on various bacteria and transforms into the cyst form when environmental conditions become unfavorable (Saygı & Polat, 2003; Sharma et al., 2000). *Acanthamoeba* species settle in human body and causes diseases such as granulomatous amebic encephalitis (GAE), cutaneous acanthamebiasis, and acanthamoeba keratitis (Mohammadi Manesh, Niyyati, Yousefi, & Eskandarian, 2014). *Acanthamoeba culbertsoni, A. Castellanii*, and *A. Rhysoides* species have been often identified in cases of granulomatous amebic encephalitis (GAE) (Saygı & Polat, 2003; Sharma et al., 2000). Acanthamoeba keratitis is a parasitosis caused by various *Acanthamoeba* species (Saygı & Polat, 2003; Sharma et al., 2000). Predisposing factors include trauma, use of contact lens, and corneal contact with contaminated water (Saygı & Polat, 2003; Sharma et al., 2000). Severe ocular pain, inflammation, visual impairment, and ring‐shaped stromal infiltration are seen in asymptomatic persons (Saygı & Polat, 2003; Sharma et al., 2000). In such cases, vision is impaired and visual loss may be seen over time (Saygı & Polat, 2003; Sharma et al., 2000). *A. Castellanii* and *A. Polyphaga* species have been often detected in patients with Acanthamoeba keratitis (Saygı & Polat, 2003; Sharma et al., 2000).

In patients with acquired immune deficiency syndrome (AIDS), infection can cause diseases such as chronic sinusitis, otitis, cutaneous lesions, sinus lesions, and skin ulcers due to the spread of the infection to various organs (Mohammadi & Niyyati, 2014; Neelam & Niederkorn, 2017; Niyyati, Lorenzo‐Morales, Rezaie, & Rahimi, 2010). Acanthamoeba infection is resistant against numerous antimicrobial agents that can be tolerated in the corneal tissue and ocular surface (Hughes, Andrew, & Kilvington, 2003; Marciano‐Cabral & Cabral, 2003; Polat et al., 2008; Tepe, Malatyali, Degerli, & Berk, 2012). It is difficult to treat and eradicate of Acanthamoeba in ophthalmic infections (Mohammadi & Niyyati, 2014; Neelam & Niederkorn, 2017). Although there are many options for the treatment of this infection, these are difficult treatment methods with limited effectiveness (Marciano‐Cabral & Cabral, 2003; Tepe et al., 2012). Effective antibiotics for the treatment include propamidine isethionate, ketoconazole, miconazole, itraconazole, and others (Ertabaklar, Dayanır, Apaydın, Ertuğ, & Walochnik, 2009). It has been proven that surgical removal of the lesion with the oral and local administration of miconazole was effective (Ertabaklar et al., 2009). In addition, hydrogen peroxide (H_2_O_2_) is a commonly used contact lens disinfectant, although is toxic for the cornea (Hughes et al., 2003). Therefore, new approaches and more efficient treatment protocols are needed for Acanthamoeba infections. Today, there are studies conducted with plant extracts and their bioactive compounds in parasitic infections, as in many other scientific areas (Derda, Hadas, & Thiem, 2009; Ródio et al., 2008; Tepe et al., 2012).

Called “çemen” in Turkish, *T. foenum‐graecum* in wild and cultivated forms is known by the names “hulba” (Arabic), “fenugreek” (English), “methi” (Hindi), “abis” (Ethiopian), and “shambala” (Armenian) (Baldemir & İlgün, 2015). The plant has been known to the public since ancient times and has been used to treat a variety of diseases (Goyal, Gupta, & Chatterjee, 2016; Yadav & Baquer, 2014). Fenugreek seeds contain steroidal sapogenin, dietary fiber, galactomannans, antioxidants, and amino acids such as 4‐hydroxyisoleucine (Ktari & Trabelsi, 2017). Due to these contents, it has sugar‐reducing, cholesterol‐reducing, fever‐reducing, anti‐inflammatory, cytotoxic, apoptosis‐activating and antifertility effects (Kassem, Al‐Aghbari, AL‐Habori, & Al‐Mamary, 2006; Pournamdari, Mandegary, Sharififar, & Zarei, 2017; Pradeep & Srinivasan, 2017; Prema & ThenmozhiA, 2016). Studies have shown its effectiveness in the treatment of diseases like obesity, diabetes, cancer, and dermatitis (Arivalagan, Gangopadhyay, & Kumar, 2013; El Bairi, Ouzir, Agnieszka, & Khalki, 2017; Khalil, Ibrahim, El‐Gaaly, & Sultan 2015; Ouzir, El Bairi, & Amzazi, 2016; Pradeep & Srinivasan, 2017). In the literature screening, there were studies reporting that *T. foenum‐graecumun* may be effective in the treatment of many diseases (Goyal et al., 2016; Ouzir et al., 2016; Yadav & Baquer, 2014). However, we could not find any study about *Acanthamoeba* species. Considering challenges in the methods that are used now to treat infections of these parasites that have quite different properties than bacteria, we aimed to investigate antiparasitic effect of methanol extract of *T. foenum‐graecum*. Accordingly, we investigated amebicidal effect of the extract on cystic form of the parasite which is reported to be more resistant than the trophozoite form.

## 2 MATERIALS AND METHODS

### 2.1 Preparation of *Trigonella foenum‐graecum* (TF) extract

Seed of TF was washed several times with deionized water and dried at room temperature. TF was powdered using a kitchen blender. 100 g of the powdered seeds was added into a 500‐ml one‐necked flask containing 250 ml methanol, and this was incubated at room temperature (RT: 25°C) for 1 day under stirring. After incubation, each solution was filtered through Whatman filter paper (No. 1) to collect the extract. This step was repeated twice using the same procedure. The extract was collected and evaporated under vacuum at 40°C and then stored at −20°C for further use.

### 2.2 Determination of the fatty acid (FA) composition of *TF*

According to the Shimadzu application catalogue, the fatty acid profiles of heat‐treated seeds of *TF* was determined. To investigate fatty acid composition, 2 seeds of *TF* samples were used for. Fat was extracted with stored in Eppendorf tubes at −20°C until analysis. The fatty acid composition was analyzed by a gas chromatography (Shimadzu GC‐2010 Plus, Japan) equipped with a Flame Ionization Detector and a 100 m × 0.25 mm ID HP‐88 column. The injector temperature was set as 250°C. The oven temperature was kept at 103°C for 1 min, then programmed from 103 to 170°C at 6.5°C/min gradient, from 170 to 215°C for 12 min at 2.75°C/min, finally, 230°C for 5 min. The carrier gas was helium with a flow rate of 2 mL/min; the split rate was 1/50. Fatty acid was defined by comparison of retention times with the known standards. The results were expressed as g fatty acid/100 g total fatty acids (Table 1)

**Table 1:** Fatty acid composition of *Trigonella foenum‐graecum* seed

Concentration,%	Fatty acid
0.11	Myristic acid methyl ester (C14:0)
0.12	Myristoleic acid methyl ester (C14:1)
10.22	Palmitic acid methyl ester (C16:0)
0.31	Heptadecanoic acid methyl ester (C17:0)
4.22	Stearic acid methyl ester (C18:0)
14.34	Oleic acid methyl ester (C18:1n9c)
45.99	Linoleic acid methyl ester (C18:2n6c)
21.35	y‐Linolenic acid methyl ester (C18:3n6)
1.24	Arachidic acid methyl ester (C20:0)
0.27	cis‐11‐eicosenoic acid methyl ester (C20:1n9)
0.47	cis‐8,11,14‐eicosatrienoic acid methyl ester (C20:3n6)
0.08	Erucic acid methyl ester (C22:1n9)
0.13	Tricosanoic acid methyl ester (C23:0)
0.14	cis‐5,8,11,14,17‐eicosapentaenoic acid methyl ester (C20:5n3)
1.00	cis‐4,7,10,13,16,19‐docosahexaenoic acid methyl ester (C22:6n3)

### 2.3 Antiparasitic activity studies

*Acanthamoeba castellanii* was taken from Cumhuriyet University Parasitology Laboratory, and *Escherichia coli* (*E. coli*) strains from Ordu University Faculty of Science and Literature Department of Biology. Parasite cultures were prepared to ensure continuation.

### 2.4 *Acanthamoeba* media

#### 2.4.1 Non‐nutrient Agar

*Escherichia coli* was proliferated on EMB medium prepared according to the procedure. Page's ameba saline solution was used in the study. The prepared solution was placed in 100 ml Erlenmeyer flasks, autoclaved at 121°C for 15 min, and stored at 4°C until use.

#### 2.4.2 Preparation of media

Agar of 1.5 g was heated and dissolved in 100 ml Page solution, autoclaved at 121°C for 15 min, and distributed to petri dishes. The prepared media were stored at 4°C until use.

#### 2.4.3 Culture

The prepared media were diluted with 0.5 ml Page solution, and 24‐hr *E.coli* strains were spread on the agar. Samples taken from *A. castellanii* strains were seeded on the media. The seeded parasites were left at 26°C for 72 hr, and the trophozoites were collected from the petri dishes without harm using Page solution and centrifuged at 1500 g for 5 min for cleaning.

To test the viability of trophozoites, 0.4% trypan blue was used and they were counted on hemocytometer slides.

### 2.5 Preparation of plant extract concentrations

Plant extract was prepared at concentrations of 32, 16, 8, 4, 2, and 1 mg/ml in 0.9% serum physiologic and distributed to sterile Eppendorf tubes at volumes of 200 μl each.

#### 2.5.1 Experimental stage

Final concentration of *Acanthamoeba castellanii* was set to 22 × 10^6^ trophozoites/ml, added to the 200‐μl tubes, and incubated at room temperature. The viability of the parasite was checked and recorded at hours 3, 24, 48, 72, 96, and 102. Tubes with no live cells identified were subjected to control seeding again, and proliferation was not observed in any of these.

Parasites not added to plant extract were left in the same environment as control.

### 2.6 Statistical analysis

The data were tested for normality using the Shapiro‐Wilk test and for homogeneity of variance using the Bartlett's test prior to the analyses. One‐way ANOVA followed by Tukey's post‐test was used to compare the groups. Descriptive statistics of the data set were expressed as means standard error of mean (*SEM*). The values of lethal doses (LD_50_ and LD_90_) were determined using probit analysis for the certain times. A *p* value ≤0.05 was considered statistically significant. All statistical analyses were performed using the SPSS v. 25 (IBM Inc., Chicago, IL, USA) statistical software.

## 3 RESULTS

**Figure 1:**
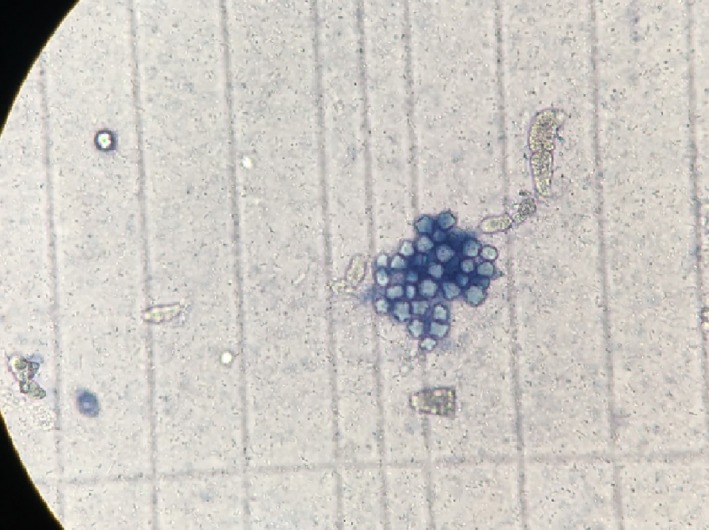
Dead *Acanthamoeba castellanii* cysts, 40X

The effect of *TF* (fenugreek) methanol extracts on *A. castellanii* is given in Table 2. Dead and live ameba are shown in Figure 1.

**Table 2:** Effect of *Trigonella foenum‐graecum* (fenugreek) methanol extracts on *Acanthamoeba Castellanii*

	Control %	1 mg/ml %	2 mg/ml %	4 mg/ml %	8 mg/ml %	16 mg/ml %	32 mg/ml %	*p*
3 hr	100.00 ± 0.00BCa	106.82 ± 6.82Aa	90.91 ± 13.64Aab	61.36 ± 15.91abc	45.45 ± 4.55bc	40.91 ± 4.55Abc	25.00 ± 11.36c	0.003**
24 hr	120.45 ± 2.27Aa	95.45 ± 4.55ABa	86.36 ± 9.09ABa	45.45 ± 13.64b	43.18 ± 2.27b	38.64 ± 2.27Ab	18.18 ± 0.00b	0.000***
48 hr	104.55 ± 0.00Ba	86.36 ± 9.09ABab	61.36 ± 15.91ABabc	47.73 ± 11.36bcd	34.09 ± 2.27cd	13.64 ± 0.00Bd	9.09 ± 0.00d	0.001**
72 hr	97.73 ± 2.27BCDa	68.18 ± 4.55BCb	54.55 ± 4.55ABbc	43.18 ± 6.82cd	29.55 ± 2.27de	11.36 ± 2.27Bef	6.82 ± 2.27f	0.000***
96 hr	93.18 ± 2.27CDa	63.64 ± 4.55BCb	47.73 ± 2.27ABbc	43.18 ± 2.27bc	27.27 ± 9.09cd	9.09 ± 4.55Bd	4.55 ± 0.00d	0.000***
102 hr	88.64 ± 2.27Da	43.18 ± 2.27Cb	34.09 ± 2.27Bbc	20.45 ± 2.27cd	20.45 ± 6.82cd	6.82 ± 2.27Bd	4.55 ± 0.00d	0.000***
*p*	0.000***	0.002**	0.032*	0.273	0.087	0.001**	0.098	

*Notes*

Horizontally, means that do not share a lowercase letter are significantly different from each other (*p* ≤ 0.05).

Vertically, means that do not share a capital letter are significantly different from each other (*p* ≤ 0.05).

Mean ± *SEM*; *Statistically significant (*p* < 0,05); **Statistically significant (*p* < 0,01); *^**^Statistically significant (*p* < 0,001).

In our study, there was a decrease in the viability of *A. castellanii*as time of exposure to *T. foenum‐graecum* extract increased, but there were no statistically significant differences between times in terms of viability rate for the doses of 4, 8, and 32 mg/ml (*p* > 0.05). Whereas in the case of *T. foenum‐graecum* extract doses lower or higher than the mentioned doses, there was a statistically significant difference in the rate of *A. castellanii* viability according to times. As seen in Table 1, there were significant decreases at the 96th hour in the control group, 72th hour at 1 mg/ml dose, and 102th hour at 2 mg/ml (*p* < 0.05). The rate of viability decreased at a shorter time with increasing the dose of *T. foenum‐graecum* extract to 16 mg/ml, and a significant decrease was observed at the 48th hour (*p* < 0.05). While there was no significant difference between 3rd and 24th hours at 16 mg/ml dose of *T. foenum‐graecum* extract (*p* > 0.05), a significant decrease occurred in the rate of viability at the 48th, 72th, and 96th compared to the 3rd and 24th hours(*p* < 0.05). In addition, a decrease by about 1/3 was seen in the viability rate after the 48th hours (*p* < 0.05). However, viability of *A. castellanii* did not change at the next hours (*p* > 0.05).

**Figure 2:**
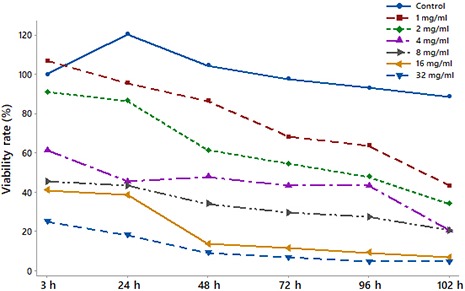
Amebicidal effect of *Trigonella foenum‐graecum* extract prepared at different concentrations on *Acanthamoeba castellanii* cysts at different hours

Amebicidal effect of *T. foenum‐graecum* extracts that were prepared in different concentrations on *A. castellanii* cysts at different hours is given in Figure** 2. When Figure 2 and Table 1 were examined, viability was decreased at all times as the dose was increased. *T. foenum‐graecum* extract significantly reduced the viability rate at the 3rd hour at a dose of 8 mg/ml, while a dose of 4 mg/ml was enough to decrease the viability rate at the 24th and 48th hours. Again, a dose of 1 mg/ml significantly reduced the viability rate at the 72th, 96th, and 102th hours (*p* < 0.05). These decreases in the viability rates occurred by about 50%.

When the table is investigated, it appears that the number of live parasites reduced compared to the control group over time. In the 1 mg/ml plant extract group, there was no clear decrease observed within the first three hours. As the dose increased in the research, there was a reduction observed in the viability of the parasite. For *TF* (fenugreek) methanol extracts above 16 mg/ml, the viability of the parasites in the 3rd hour had a reduction of 50% or more. In the 24th hour, the parasite viability was ≤50% in the ≥2 mg/ml concentration groups, while in the 48th, 72nd, and 96th hour, the parasite viability was ≤50% in ≥2 mg/ml concentration groups. In the 102th hour, the parasite viability was ≤50% for all plant extract groups including 1 mg/mL concentration.

In this study also investigated that the LD50 and LD90 value of *T. foenum‐graecum* extract on *A. castellanii* cysts and trophozoites. (Table 3). When the table was examined, LD50 and LD90 values were determined as 36.92 and 5.09 mg/ml at the 3rd hour, respectively. In addition, the values of LD50 and LD90 were found as 0.30 and 16.42 mg/ml at 102th hour, respectively.

**Table 3:** LD50 and LD90 value at certain time against concentration of *Acanthamoeba castellanii*

Time	LD_50_ (95% confidence limit)	LD_90_ (95% confidence limit)
3 hr	15.09^a^ (8.38–27.79)	36.92^a^ (25.46–82.82)
24 hr	12.26^a^ (5.59–22.11)	34.07^a^ (23.52–75.68)
48 hr	6.19^a^ (0.15–11.02)	23.99^a^ (17.14–44.68)
72 hr	5.71^a^ (1.25–10.12)	20.61^a^ (14.61–38.21)
96 hr	4.47^a^ (0.27–8.16)	18.30^a^ (12.99–33.66)
102 hr	0.30^a^ (0.00–4.77)	16.42^a^ (10.43–45.93)

*Note*

a mg/ml.

## 4 DISCUSSION

Effective treatment of *Acanthamoeba*‐linked central nervous system infections generally involves combined treatment beginning in the early stage of infection. Additionally, it is known that the majority of therapeutic agents are not effective at later stages of the infection. Generally, combined chemotherapy appears to be more successful than single medication treatments. Therapeutic agents used routinely include a combination of medications like ketoconazole, fluconazole, itraconazole, pentamidine isethionate, azithromycin, sulfadiazine, amphotericin B, rifampicin, voriconazole, and miltefosine (Webster, Umar, Kolyvas, & Bilbao, 2012). Due to low treatment efficiency, it is reported that combinations of the medications listed above improve less than 10 granulomatous amebic encephalitis patients (Walochnik, AichelburgA, & Steuer, 2008). For Acanthamoeba keratitis (AK) treatment, a combination of chemotherapeutic agents like polyhexamethylene biguanide which destroys cell membranes and propamidine isethionate which inhibits DNA synthesis are commonly used (Hargrave & McCulley, 1999; Khojasteh, Niyyati, Rezaei, & Mohebali, 2014). Additionally, chlorhexidine is used for AK treatment alone or with other medications (Arnalich‐Montiel & Almendral, 2012; Kosrirukvongs, Wanachiwanawin, & Visvesvara, 1999). However, there have been Acanthamoeba strains reported with bad cysticidal or even resistance to propamidine (Ficker, Seal, Warhurst, & Wright, 1990; Lorenzo‐Morales & Martín‐Navarro, 2013). In situations with permanent infection and inflammation, corticosteroids may be used; however, it is reported that their use may cause suppression of the patient's immunologic response. Corticosteroids inhibit the encystment and excision processes of Acanthamoeba, and it may also lead to the development of resistance in Acanthamoeba (Lorenzo‐Morales, Martín‐Navarro, LópezArencibia, & Santana‐Morales, 2010). These combination treatments normally are active against the trophozoite stage, so negative aspects include that Acanthamoeba cysts may remain viable and severe and repetitive keratitis may occur. Further, resistance of double‐walled cysts is due to cellulose molecules found in the inner layer of the cysts. Additionally, the majority of the medications above are very toxic for human keratocytes. The treatment duration for these medications is also very long (may last up to 6 months) (Niyyati et al., 2010; Reinhard & Sundmacher, 2000). Generally, deficiencies of the reported and indicated effective chemotherapeutic agents have lead researchers in the field to give high priority to new compounds for Acanthamoeba infections. In this way, there is a trend of reporting naturally sourced compounds (mainly isolated from plants and herbs) rather than chemical medications (Shinwari, 2010).

Plants produce antimicrobial materials to protect themselves from the pathogenic effects of microorganisms. The main chemical components of fenugreek seed comprise polysaccharides, flavonoids, saponins, fixed oils, and some alkaloids (trigonelline, choline). At the same time, the fenugreek seeds are rich in iron, calcium, phosphorus, and vitamins. Due to the large amounts of galactomannan found in the endosperm of the seed, the plant is thought to increase lactation. Additionally, due to its active components, it has many types of pharmacological activities. Immunomodulatory, anticancer, antidiabetic, gastroprotective, anti‐inflammatory, and antipyretic properties of the plant have been identified. Many studies have shown that fenugreek contains defensin (Baldemir & İlgün, 2015; Toppo, Akhand, & Pathak, 2009). Defensin and defensin‐like proteins are antifungal proteins and are found in abundant amounts in the seed to protect against soil fungi (Karri & Bharadwaja, 2013; Oddepally & Guruprasad, 2015; Olli & Kirti, 2006).

Some studies have shown that fenugreek seeds are anti‐cancerogenic. They are reported to kill cancer cells from human colon, osteosarcoma, leukemia, lung, and liver cancers. This effect prevents cell growth and is stated to begin apoptosis in a dose‐dependent manner (Alsemari et al. 2014).

In the present study, we found a significant difference between times of exposure to *T. foenum‐graecum* extract and doses of the plant extracts in terms of parasite viability (*p* < 0.05). This may be interpreted as that although changing by extract doses, the rate of viability decreased as the dose increased. Again, a decrease was observed in the viability depending of times of exposure to the extract. In addition, variations in the levels of parasite viability may be explained by different rates of antiparasitic effects of extract concentrations.

In this study, the viability rate for the parasite in *TF* methanol extract in the 3rd hour was about LD50 for concentrations of 15.09 mg/ml and LD90 for concentrations of 36.92 mg/ml. Again in the 24th hour, the viability rate was LD50 for concentrations of 12.26 mg/ml and LD90 for concentrations of 34.07 mg/ml, and again in the 48rd hour was about LD50 for concentrations of 6.19 mg/ml and LD90 for concentrations of 23.99 mg/ml. While the viability rate of 72th and 96th hour was LD50 for all concentrations of, respectively, 5.71 mg/ml and 4.47 mg/ml, and LD90 for all concentrations of, respectively, 20.61 and 16.42, the viability rate of 102th hour was LD50 for all concentrations of 0.30 mg/ml and LD90 for concentrations of 16.42 mg/ml.

In the available literature, there was no study on the amebicidal effect of fenugreek methanol extracts. The study by Dodangeh et al. prepared chloroform fractions from *TF* seed (10, 15, 20 and 25 mg/ml dilutions) and researched the anti‐acanthamoeba activity and determined the toxicity of these fractions on mouse macrophage cells with the MTT methods (25, 50, 100, 200, 300, 400, 500 mg/ml). Accordingly, when trophozoites/cysts were incubated for 24 hours in 15 and 20 mg/ml concentrations with the remaining chloroformic fraction, they were destroyed. The viability of macrophage cells was recorded as 100% for 25 and 50 mg/ml concentrations with chloroformic fraction. The results showed that the plant fractions were safe for mammalian cells. In this study, the effect of methanol extracts (1, 2, 4, 8, 16, 32 mg/ml concentrations) prepared from seed of *A. castellanii* was investigated. It was observed that number of live parasites reduced compared to the control group over time (Dodangeh, Niyyati, & Kamalinejad, 2001).

In different studies, the amebicidal effects of plant extracts such as *Thymus sipyleus subsp. Sipyleusvar.sipyleus* have been reported (Polat, Vural, Tepe, & Cetin, 2007) at 32.0 mg/ml dose, *Allium sativum* at 3.9 mg/ml (Malatyali & Tepe, 2012; Malatyali, Tepe, Degerli, & Berk, 2012), *T. foenum‐graecum* at 400 mg/ml (Dodangeh et al., 2001), *Peucedanum caucasicum, P. palimbioides, P. chryseum, and P. longibracteolatum* at 32 mg/ml (Malatyali et al., 2012), *S. staminea* at 16 mg/ml (Goze, Alim, Dag, & Tepe, 2009), *Propolis* at 15.62 mg/ml (Topalkara, Vural, Polat, & Toker, 2007), *Origanumsyriacum* and *Origanumlaevigatum* at 32.0 mg/ml (Degerli, Tepe, Celiksoz, & Berk, 2012), and *Buddleia cordata* at 32 mg/ml (Rodríguez‐Zaragoza, Ordaz, Avila, & Muñoz, 1999). Additionally, it is reported that the doses used were not toxic. Karakuş et al. investigated the amebicidal activity of maleic anhydride‐co‐vinyl acetate (MAVA) on Acanthamoeba trophozoites and cysts and identified that in the first 48 hr, there was a rapid reduction in the proportion and number of live trophozoites, except for the 32.0 mg/ml dose. The highest MAVA dose suppressed proliferation of trophozoites within 3 hr. They stated that cysts were more resistant to this suppressive effect (Karakuş, Malatyalı, Zengin, & Değerli, 2013). Again, the minimal inhibitory concentrations (MIC) of *Arachishypogaea* L.*, Curcuma longa* L., and *Pancratium maritimum* L., *A. hypogaea* of 100 mg/ml were checked at 24, 48, and 72 hr and *C. longa* was effective at 1 mg/ml in 48 hr, while *P. maritimum* was effective at 200 mg/ml in 72 hr (El‐Sayed, Ismail, Ahmed, & Hetta, 2011).

Sulieman et al. investigated the fatty acid composition of AF seeds (12:0, lauric acid; 14:0, myristic acid; 16:0, palmitic acid; 16:1, palmitoleic acid; 18:0, stearic acid; 18:1, oleic acid; 18:2, linoleic acid; 18:3, linolenic acid; 20:0, arachidic acid; 20:1, eicosapentaenoic acid; 22:0, behenic acid; 24:0, lignoceric acid) and total fat content (8.4%) in their study (Sulieman, Ali, & Hemavathy, 2008). In our study, very close values were obtained for the main components detected in *T. foenum‐graecum* oil (linoleic acid, y‐linolenic acid, oleic acid, palmitic acid, stearic acid) and the percentage concentrations of these components (Table 2)

Hemavathy and Prabhakar found that the total lipid content of *T. foenum‐graecum* seed was composed of 84.1% neutral lipids, 5.4% glycolipids, and 10.5% phospholipids. Neutral lipids have been shown to contain mostly triacylglycerols (86%), diacylglycerols (6.3%) and small amounts of monoacylglycerols, free fatty acids, and sterols (Hemavathy & Prabhakar, 1989).

There are studies in the literature reporting that linoleic acid and y‐linolenic acid are effective on mitochondria of *A. castellanii* (Czarna & Jarmuszkiewicz, 2005; Jarmuszkiewicz, Sluse‐Goffart, Hryniewiecka, & Sluse, 1999; Sayanova et al., 2006). In this study, linoleic acid methyl ester (45.99%) and y‐linolenic acid (21.35%) were identified as the highest percentages of main components of *T. foenum‐graecum* seeds. Moreover, studies in the literature have shown that *T. foenum‐graecum* seed contained some active components, such as alkaloids, flavonoids and polysaccharides, phenolic acids, triterpenoids, nicotinic acid, and steroidal sapogenins (Benayad, Gómez‐Cordovés, & Es‐Safi, 2014; Huang & Liang, 2000; Kenny, Smyth, Hewage, & Brunton, 2013; Rayyan, Fossen, & Andersen, 2010; Sauvaire, Ribes, Baccou, & Loubatieerres‐Mariani, 1991; Shang et al., 1998). It is believed that the strong effect of *T. foenum‐graecum* seed extracts on *A. castellani* proliferation is due to the combination of these main components.

## 5 CONCLUSION

This study determined that *T. foenum‐graecum* prevented the proliferation of the parasite at certain times. In addition, we thought that the dose can be increased when a rapid effect of *T. foenum‐graecum* extract on the parasite is desired (LD90 = 36.92 mg/ml), and the dose can be decreased if a long‐term effect is expected (LD90 = 16.42 mg/ml) (Table 3). However, further in vivo and controlled experimental studies are needed in order to find out how to use this plant as medication.

## CONFLICT OF INTEREST

The authors declare no conflict of interest.

## ETHICAL STATEMENT

Human testing and animal testing were not necessary in this study.
